# Characterization and performance evaluation of the XSPA-500k detector using synchrotron radiation

**DOI:** 10.1107/S1600577520016665

**Published:** 2021-02-10

**Authors:** Yasukazu Nakaye, Takuto Sakumura, Yasutaka Sakuma, Satoshi Mikusu, Arkadiusz Dawiec, Fabienne Orsini, Pawel Grybos, Robert Szczygiel, Piotr Maj, Joseph D. Ferrara, Takeyoshi Taguchi

**Affiliations:** a Rigaku Corporation, 3-9-12 Matsubara-cho, Akishima-shi, Tokyo 196-8666, Japan; b Detector Group, Synchrotron SOLEIL, L’Orme des Merisiers, Saint Aubin – BP 48, Gif-sur-Yvette 91192, France; c Department of Measurement and Electronics, AGH University of Science and Technology, al. Mickiewicza 30, Krakow 30-059, Poland; d Rigaku Americas Corporation, 9009 New Trails Drive, The Woodlands, TX 77381, USA

**Keywords:** single photon counting, hybrid pixels, synchrotron radiation

## Abstract

Hybrid photon counting (HPC) detectors are widely used at both synchrotron facilities and in-house laboratories. The features of HPC detectors, such as no readout noise, high dynamic range, high frame rate, excellent point spread function, no blurring *etc.* along with fast data acquisition, provide a high-performance detector with a low detection limit and high sensitivity. Several HPC detector systems have been developed around the world. A number of them are commercially available and used in academia and industry. One of the important features of an HPC detector is a fast readout speed. Most HPC detectors can easily achieve over 1000 frames s^−1^, one or two orders of magnitude faster than conventional CCD detectors. Nevertheless, advanced scientific challenges require ever faster detectors in order to study dynamical phenomena in matter. The XSPA-500k detector can achieve 56 kframes s^−1^ continuously, without dead-time between frames. Using ‘burst mode’, a special mode of the UFXC32k ASIC, the frame rate reaches 1 000 000 frames s^−1^. XSPA-500k was fully evaluated at the Metrology beamline at Synchrotron SOLEIL (France) and its readout speed was confirmed by tracking the synchrotron bunch time structure. The uniformity of response, modulation transfer function, linearity, energy resolution and other performance metrics were also verified either with fluorescence X-rays illuminating the full area of the detector or with the direct beam.

## Abstract 2

A Rigaku XSPA-500k detector was evaluated and its performance characterized on the Metrology beamline at Synchrotron SOLEIL together by the SOLEIL Detector Group, AGH-UST and Rigaku.

## 1. Introduction   

Hybrid photon-counting (HPC) detectors for X-ray applications have been in development for more than three decades. Many research institutes and universities have developed various types of HPC detectors. Some of those are commercialized and distributed to academic laboratories and industrial facilities. Nowadays, many advanced X-ray techniques cannot be performed without an HPC detector. The most advanced experiments in fast dynamics, such as pump–probe experiments and X-ray photon correlation spectroscopy (XPCS), require a very fast detector to study short time scale phenomena in complex condensed matter. Rigaku and AGH-UST developed an HPC detector and began distributing this detector and its variants all over the world in 2014 (Maj *et al.*, 2014 ▸). Since then, Rigaku and AGH-UST developed a new, much faster detector capable of a 56 000 frames s^−1^ data rate with a very uniform detection area. This detector, the XSPA-500k, was evaluated and its performance characterized on the Metrology beamline at Synchrotron SOLEIL together by the SOLEIL Detector Group, AGH-UST and Rigaku.

## 2. XSPA-500k detector   

**Figure 1:**
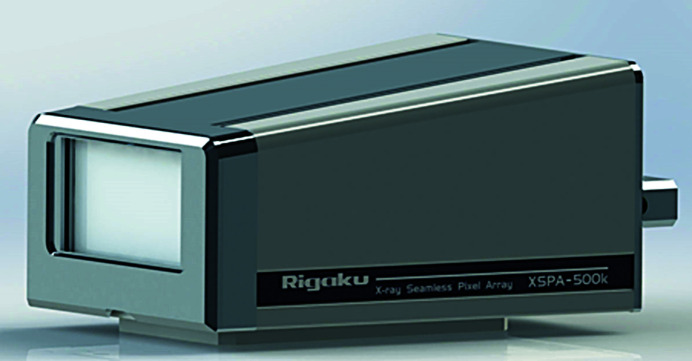
XSPA-500k detector.

The XSPA-500k detector is an HPC detector (Fig. 1 ▸) with 524k (1024 × 512) 76 µm × 76 µm pixels over a 77.8 mm × 38.9 mm detection area. The detector consists of one large, unique monolithic silicon sensor and 16 UFXC32k application-specific integrated circuits (ASIC). The details of the UFXC32k ASIC will be described in the next section. The sensor and ASICs are flip chip bonded with small indium metal bumps, that is, using a standard process for this kind of detector. However, the size of all pixels in the XSPA-500k is the same. Most HPC detectors with a detection area larger than 25 mm × 25 mm that are built with more than one ASIC have larger pixels at the boundaries of the ASICs. Those larger pixels are necessary in order to compensate for the physical gaps between ASICs. X-ray events observed in those larger pixels are numerically processed or divided into virtual pixels of equal size. Depending on the detector, the size of the boundary pixels is 1.5 to 3 times larger in one direction and 2.25 to 9 times larger in area than a normal pixel (Kraft *et al.*, 2009 ▸; Cudié, 2007 ▸). As X-ray events are distributed numerically, spatial information is biased and the statistics do not represent the actual X-ray events on those pixels. These larger pixels reach the counting limitation faster as they accumulate more X-ray photons than normal pixels and present more charge drifted to the same circuit. Therefore, a user who wishes to execute a very accurate experiment usually masks out those pixels and does not use them in the analysis. In order to address this issue, Rigaku developed the X-ray seamless pixel array (XSPA) detector using cutting-edge technology (Sakamura *et al.*, 2019 ▸), which provides equal-size pixels over the entire detection area. In other words, all the pixels have the same characteristics and the entire array can be used for analysis. The XSPA-500k is a single-module detector with dimensions 150 mm (W) × 100 mm (H) × 208 mm (D). Energy thresholds can be set from 2 keV to 30 keV for this detector. A 320 µm-thick silicon sensor was used for this work, but other thicknesses and materials, such as GaAs, CdTe and CZT, can also be used.

### 2.1. UFXC32k readout chip   

**Figure 2:**
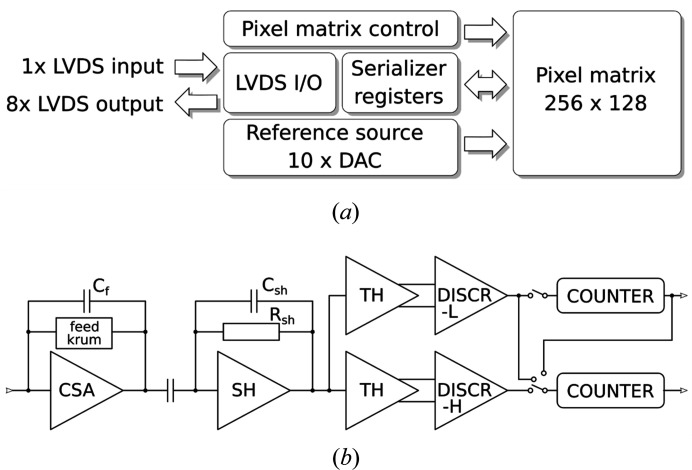
UFXC32k chip: (*a*) simplified block diagram of the chip, (*b*) simplified block diagram of a single pixel.

The UFXC32k integrated circuit (ultrafast X-ray chip with 32k channels) fabricated using CMOS 130 nm technology, has the dimensions 9.64 mm × 20.15 mm and contains approximately 50 million transistors (Grybos *et al.*, 2016 ▸). The core of the integrated circuit is a matrix of 128 × 256 pixels and 75 µm × 75 µm in size [Fig. 2 ▸(*a*)]. Each pixel operates in single-photon-counting mode with two energy thresholds working independently. Each pixel contains a charge-sensitive amplifier (CSA) with a Krummenacher feedback discharge circuit (feed Krum), an amplifier (SH, with a minimum peaking time of 40 ns), threshold setting blocks (TH), two discriminators (DISCR) and two 14-bit ripple counters (COUNTER) [Fig. 2 ▸(*b*)]. The possibility of tuning the current in the CSA feedback discharge block (feed Krum) allows for a compromise between the noise and count-rate performance of the UFXC32k readout pixel required by a given application (Kmon *et al.*, 2016 ▸).

The energy threshold levels are set in common for all the readout channels in the pixel matrix and are adjusted depending on the applied X-ray energy and the application requirements. To minimize the effective threshold spread from pixel to pixel, each pixel contains two independently working trimming DACs at the discriminator inputs. Each pixel also has the possibility to independently trim the gain in the analog front-end. In addition, the analog front-end electronics allow processing sensor signals of both polarities (holes or electrons) and, as a result of effective detector leakage current compensation, it is possible to use the UFXC32k IC with different sensor materials.

During the data readout phase, the counters in each column form a shift register. The data from the register are loaded, bit by bit, into the peripheral fast 128-bit registers and shifted out of the UFXC32k chip via eight low-voltage differential signal (LVDS) parallel lines. The data readout in the chip can operate in several different modes, the main modes being:

(i) Normal mode with two 14-bit counters connected to the outputs of discriminators DISCR-L and DISCR-H.

(ii) Long-counter mode with one 28-bit counter connected to the output of discriminator DISCR-A.

(iii) Continuous mode whereby one of the 14-bit counters is connected to discriminator DISCR-L and the other forms a shift register in the chip column and sends the data out (or vice versa). One can select the number of bits read out from each pixel to optimize the UFXC32k frame rate; for 2-bit frames this is more than 50 kHz.

(iv) Burst mode consisting of 13 2-bit frames at a rate up to 1.2 Mframes s^−1^ for single-chip operation with a bus clock frequency of 320 MHz.

### 2.2. Frame rate   

**Figure 3:**
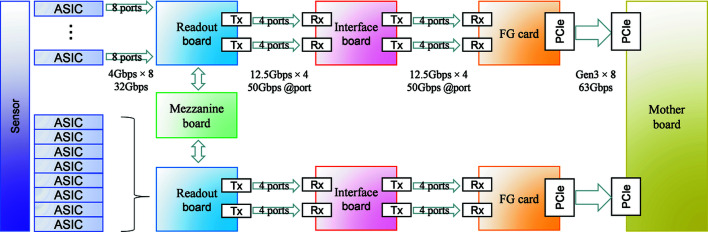
XSPA-500k detector system block diagram.

The detector front-end is connected to two mezzanine readout boards with eight ASICs each (Fig. 3 ▸). The mezzanine readout boards are controlled by one board. Data from each ASIC are read out and transferred to an FPGA via eight ports on double-data-rate (DDR) at 250 MHz bus clock frequency. Therefore, the data transfer rate of each ASIC is 4 Gb s^−1^. A half-module has eight ASICs and sends 32 Gb of data to the FPGA in 1 s. Data from the detector are transferred through an optical cable. A mezzanine board is the transmitter and a server PC with a frame grabber board is the receiver. As data are transferred using 8b/10b encoding, 524 288 byte half module data with 16-bit depth (14-bit counter) become 629 146 bytes. For 2-bit depth readout, the data size of a single frame is 65 536 bytes and becomes 78 644 bytes. The data transfer rate of the transmitter is 12.5 Gb s^−1^. Four transmitters are used for one data port and a maximum of 50 Gb of data can be transferred in 1 s. Although the data size is about two-thirds of the maximum capacity of the transmitter, we measured the real bandwidth of the PCIe gen3 ×8 to be about 30 Gb s^−1^ (the theoretical bandwidth is about 63 Gb s^−1^) and this limits the maximum data transfer rate of the system. According to this, using the zero dead-time (ZDT) mode, or switching the counter for recording and reading at the same time, 8500 frames of 16-bit depth (14-bit counter) data can be read in 1 s without a time gap between frames. By reducing the reading bit depth, a higher frame rate can be achieved. Reading 2-bits pixel^−1^ with ZDT mode, a rate as fast as 56 000 frames s^−1^ is achieved. Using burst mode, that is, recording several frames of data in the counters at 2-bits pixel^−1^, a total of 13 frames at 970 000 frames s^−1^ is possible. The properties of the XSPA-500k are unique and the authors decided to complete a battery of tests to determine the limits of the detector.

## 3. Detector performance evaluation using synchrotron radiation   

The detector was developed at Rigaku and tested with a conventional X-ray generator at BL26B2 and BL29XU, SPring-8. It has also been used for an X-ray photon correlation spectroscopy (XPCS) experiment at the Advanced Photon Source (APS) (Zhang *et al.*, 2021 ▸). However, the complete detector performance has only been evaluated by the SOLEIL Detector Group at the Metrology beamline in different acquisition modes, as described in the following sections.

### 3.1. Experimental setup   

**Figure 4:**
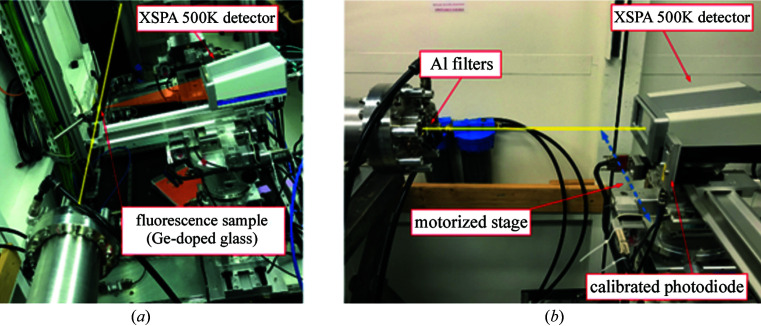
Experimental configurations on the Metrologie beamline used to evaluate the performance of the XSPA-500k detector: (*a*) fluorescence setup with Ge sample placed in the direct beam path and the detector on the perpendicular axis, (*b*) direct-beam setup with the detector placed on the direct beam attenuated with a series of aluminium foils.

The complete characterization of the XSPA-500k detector was carried out on the hard X-ray branch of the Metrology beamline at Synchrotron SOLEIL (Idir *et al.*, 2010 ▸). Two experimental configurations were used to evaluate the detector performance, *i.e.* fluorescence and direct-beam setups. The fluorescence setup [Fig. 4 ▸(*a*)] was used to assess the homogeneity and spatial properties of the detector. The fluorescence target, a Ge-doped glass, was located in the direct beam path (12 keV) under a 45° angle and with the detector on the perpendicular axis to obtain a uniform illumination over the whole surface with 9.9 keV photons (Ge *K*α line). The direct-beam setup [Fig. 4 ▸(*b*)] was used to evaluate the performance of the detector at high photon count rates. In this configuration the detector was exposed to a direct monochromatic beam with an energy of 9.9 keV. The intensity of the beam was changed with a series of aluminium foils located at the beam exit. The beam flux was measured with a calibrated photodiode placed at the same distance as the XSPA-500k camera. During all measurements, the synchrotron was operated in hybrid filling mode, *i.e.* with an isolated electron bunch of 5 mA in the middle of one-quarter of the storage ring orbit and multi-bunches of about 1.5 mA in the remaining three-quarters of the orbit.

### 3.2. Test beam results   

#### 3.2.1. Flat-field corrections and signal-to-noise ratio   

**Figure 5:**
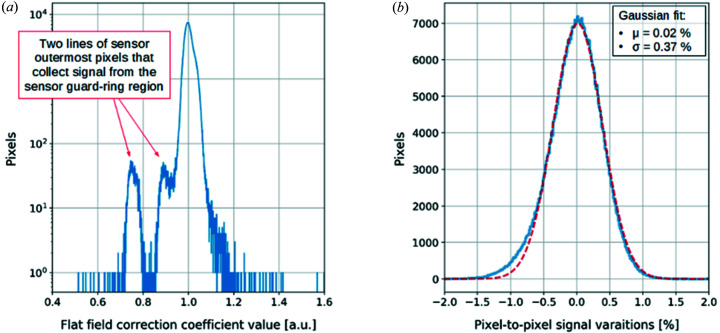
Histograms of the (*a*) flat-field correction coefficients for images acquired at 9.9 keV energy and (*b*) the pixel-to-pixel variation after applying the flat-field correction.

Flat-field correction is a technique that allows for the removal of fixed-pattern noise (FPN) from a raw image due to the residual threshold dispersion in the readout ASICs and inhomogeneities in the sensor, therefore increasing the Poisson limited range of the detector. To efficiently apply the flat-field method, the correction coefficients should be obtained from a raw and uniform image acquired at the same energy as images to which this correction will be applied, with significantly higher statistics (Janesick, 2007 ▸). For the XSPA-500k detector, the flat-field correction coefficients have been calculated from the image acquired with Ge fluorescence at 9.9 keV with the mean number of counted photons of about 5 × 10^5^ per pixel. The resulting histogram of the obtained correction coefficients is shown in Fig. 5(*a*) ▸. Most pixels have a correction coefficient of about 1 (normalized value), represented by the highest peak on the histogram; however, two other distinct peaks can be observed. These two peaks correspond to the two outermost lines of sensor pixels that collect signal from the sensor guard-ring region, with the result that they appear slightly larger in area than the other pixels. The resultant flat-field correction table has been applied to all data acquired at 9.9 keV energy. The resulting pixel-to-pixel signal variations, after applying flat-field corrections, are reduced to σ = 0.37% which is a very low value and within ±1% range for almost all pixels [see Fig. 5(*b*) ▸].

**Figure 6:**
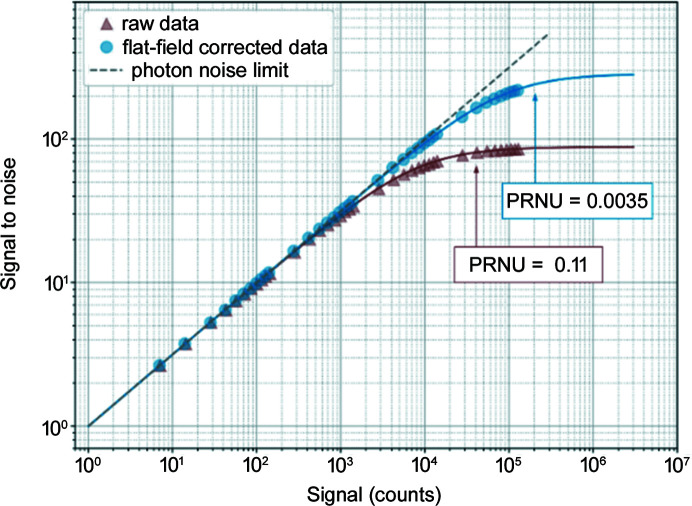
Signal-to-noise plot for raw and flat-field corrected images.

Another important parameter is the signal-to-noise ratio (SNR) as a function of the signal, as shown in Fig. 6 ▸. This SNR curve directly provides the Poisson limited range of the detector (Medjoubi & Dawiec, 2017 ▸). Because hybrid single-photon-counting detectors do not suffer from readout noise, only two distinct remaining noise regimes are observed in the SNR curve: Poisson noise (shot noise) and fixed-pattern noise. The boundary between the two noise sources is determined by the photon response non-uniformity (PRNU) with the Poisson noise limit at 1/PRNU^2^. The PRNU is obtained by fitting the analytical model to the experimental data. It is clearly shown that the flat-field correction increases the Poisson range limit by a factor of 10, from ∼10^4^ photons per pixel up to ∼10^5^ photons per pixel.

Note that bad pixels were masked offline and not included in any analysis presented here. These pixels were identified from an averaged series of flat-field images and the value of every pixel was compared with the local median value (50 × 50 pixels). Any pixel with a signal that is less than half of the median or greater than twice the median was considered to be a bad pixel and was discarded from the analysis. The total number of pixels identified as bad on the XSPA-500k detector amounts to 0.02% of all pixels, which represents a very low number of pixels.

#### 3.2.2. Energy resolution assessment   

**Figure 7:**
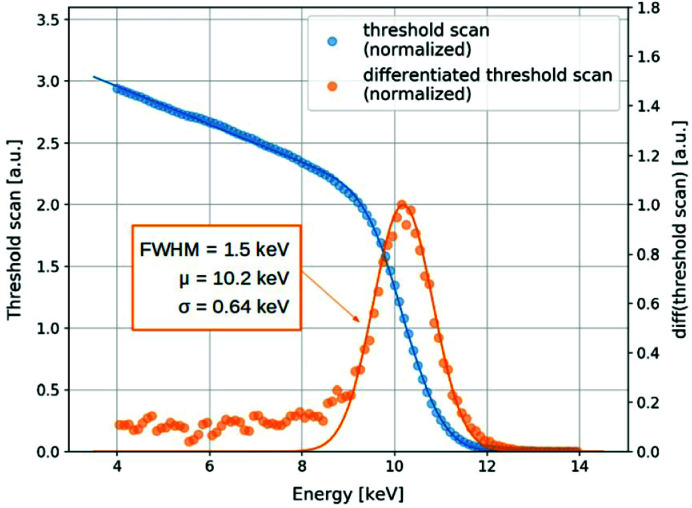
Averaged threshold scan for a complete detector measured at 9.9 keV (blue plot) and the resulting energy spectrum obtained by differentiation of the scan data (orange plot).

A narrow energy resolution range in a detector is essential for accurate discrimination of detected photons as a function of their energy. It is particularly important for systems with several thresholds, such as the XSPA-500k. The energy resolution of the complete detector has been measured by performing a threshold scan with 9.9 keV fluorescence photons from a Ge glass. The scan was carried out with threshold values varying from 4 keV to 18 keV in steps of 100 eV. For every threshold value, a single image was acquired and averaged. The resulting integral spectrum (so-called S-curve) is shown in Fig. 7 ▸, as a blue curve together with a fitted model function. The data were normalized with the number of counts at the inflection point of the S-curve. The energy resolution was derived directly from the differentiated threshold scan as the full width at half-maximum (FWHM) of the corresponding energy peak. The resulting differential curve is fitted with a Gaussian function to the energy peak, as shown in Fig. 7 ▸ by the orange curve. The measured energy resolution for the entire detector is about 1.5 keV. The values obtained are typical for a single-photon-counting detector and comparable with the state-of-the-art detectors (Ballabriga *et al.*, 2016 ▸).

#### 3.2.3. Imaging performance   

**Figure 8:**
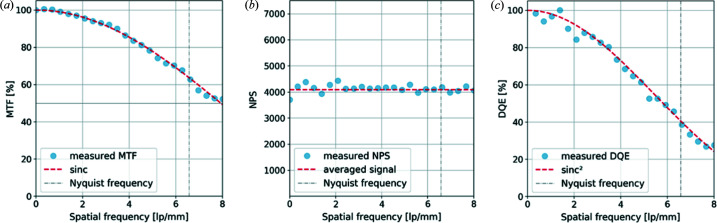
Figures of merit describing the imaging performance of the XSPA detector system measured at 9 keV: (*a*) MTF, (*b*) NPS and (*c*) DQE. The vertical dashed lines on all plots represent the Nyquist frequency of the detector, and measure 6.58 line-pairs mm^−1^ for a pixel pitch of 76 µm.

The imaging performance of the XSPA detector has been assessed by measuring the standard figures of merit: the modulation transfer function (MTF), noise power spectrum (NPS) and detective quantum efficiency (DQE). The MTF is a performance metric that describes the transfer of the incident contrast through the detector system as a function of spatial frequency. To determine the MTF, the slanted-edge method described by Samei *et al.* (1998 ▸) was followed. A thin and sharp gold foil, tilted with respect to a pixel axis, was imaged with a direct 9.9 keV beam. The oversampled edge-spread function (ESF) was obtained by projection and realignment of pixel counts from several consecutive lines and differentiated to obtain the line-spread function (LSF). The pre-sampled MTF was calculated by the discrete Fourier transform of the LSF. The resultant MTF [Fig. 8 ▸(*a*)] follows the curve of an ideal detector described by the sinc function and reaches the expected 63% at the Nyquist frequency, which is 6.58 line-pairs mm^−1^ for a pixel pitch of 76 µm.

The NPS provides information as to whether the number of counts from different pixels is correlated or not. To calculate the NPS, two flat-field images were acquired at 9.9 keV, subtracted and normalized by 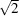 to remove the incident beam shape and FPN (Ponchut *et al.*, 2005 ▸). The 2D NPS was calculated from several regions of 200 × 200 pixels and the 1D NPS was obtained by angular integration of the 2D NPS. The resulting 1D NPS [Fig. 8 ▸(*b*)] displays a white spectrum, indicating that there is no correlation between the signal measured by different pixels. The last figure of merit, the detective quantum efficiency (DQE), can be calculated directly from the MTF and NPS measurements. In the spatial domain DQE is expressed as
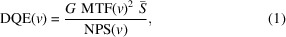
where *G* is the conversion factor (or gain) and 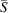 is the average signal in the region of interest (ROI) over which the NPS was calculated (Ponchut *et al.*, 2005 ▸). The measured DQE [Fig. 8 ▸(*c*)] at zero spatial frequency reaches a quantum efficiency limited by the absorption limit of the silicon sensor.

**Figure 9:**
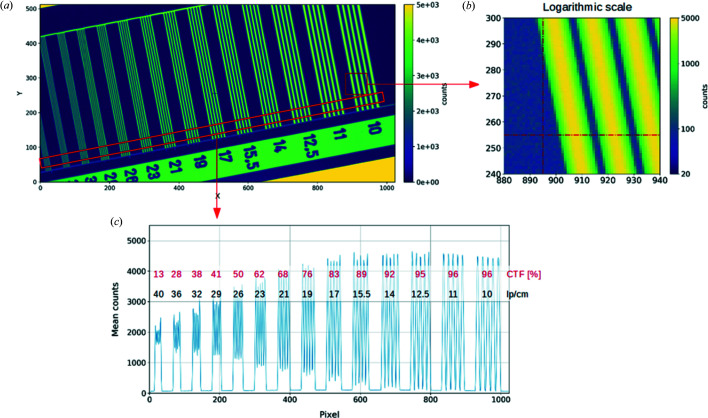
(*a*) Image of the bar pattern acquired with Ge fluorescence (9.9 keV). (*b*) Small ROI covering the area of the four adjacent readout chips (dashed lined). (*c*) Bar pattern profile with corresponding CTF values.

Further assessment of the imaging performance of the detector was completed by the acquisition of lead resolution pattern images with a 9.9 keV beam (Ge fluorescence), shown in Fig. 9 ▸(*a*). The bar pattern is composed of 0.01 mm-thick lead foil encapsulated in a transparent plastic housing. Flat-field and bad-pixel corrections were applied to the image. It is worth noting the quality and homogeneity of the image. As a result of the gapless sensor design, which does not have larger sensor pixels at the boundaries of readout chips, virtual pixel correction is not necessary. A small-image ROI covering an area of four adjacent readout chips is shown in Fig. 9 ▸(*b*) with a logarithmic scale in order to enhance the low-counts region. The boundaries of the readout chip are highlighted with red dashed lines. The intensity profiles obtained from the highlighted ROI is shown in Fig. 9 ▸ along with the contrast transfer function (CTF) values in this configuration. The CTF calculation presented in this work is only demonstrative as the parallax effect due to the isotropic beam as well as diffusion from the plastic housing were neither corrected for nor taken into account.

#### 3.2.4. Count rate linearity   

**Figure 10:**
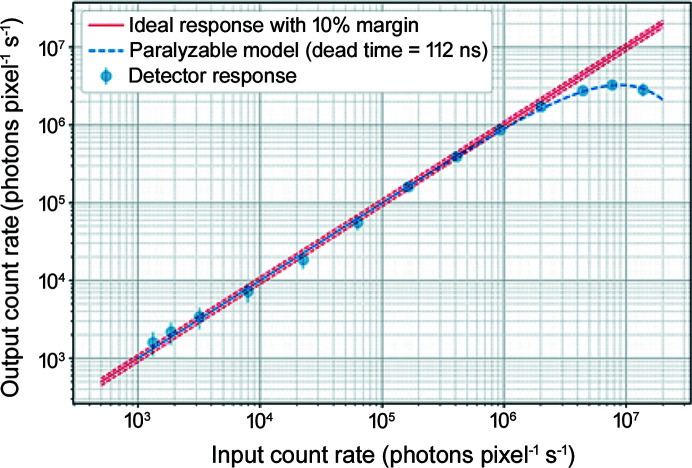
High count rate performance linearity of the detector with a fitted paralyzable model. The linear limit is estimated based on the fitted dead-time value and can reach 9.4 × 10^5^ photons s^−1^ pixel^−1^.

The count rate limit of the detector was measured using the direct-beam configuration shown in Fig. 4 ▸(*b*). As discussed in Section 2.1, the detector can have several different front-end settings which determine its main performance parameters such as noise, energy resolution and count rate. During the measurement, the detector was configured to so-called ‘normal’ mode with rather low CSA feedback current [as a trade-off between noise and pulse width at CSA output (Kmon *et al.*, 2016 ▸)] and, therefore, represents only a fraction of the full count-rate capabilities of the detector. During the measurement, a region of 35 × 36 pixels (∼2.6 mm × 2.7 mm) was illuminated with a direct beam of 9.9 keV energy. The direct beam non-uniformity has been measured and is at a level of 7%. The intensity of the beam was changed by means of 12 aluminium foils of 125 µm thickness placed in the beam path. For each beam flux value, *i.e.* number of foils in the beam path, a reference measurement with a calibrated silicon photodiode (51 µm thickness) was performed in order to accurately estimate the total number of photons in the spot. Both the XSPA detector and the photodiode were located at the same distance from the beam exit window. The number of photons per pixel was obtained by averaging the total signal with the number of illuminated pixels. The measured count rate of the detector is shown in Fig. 10 ▸.

Measured data were fitted with a paralyzable detector model (Knoll, 2000 ▸) function, described as
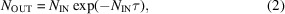
where *N*_OUT_ is the measured output count rate, *N*_IN_ is the input photon rate and τ is the detector dead-time. The measured dead-time is 112 ns, hence the maximum input photon count rate can be as high as 9 × 10^6^ photons pixel^−1^ s^−1^ corresponding to 1.6 × 10^9^ photons s^−1^ mm^−2^.

#### 3.2.5. Short gate time performance   

**Figure 11:**
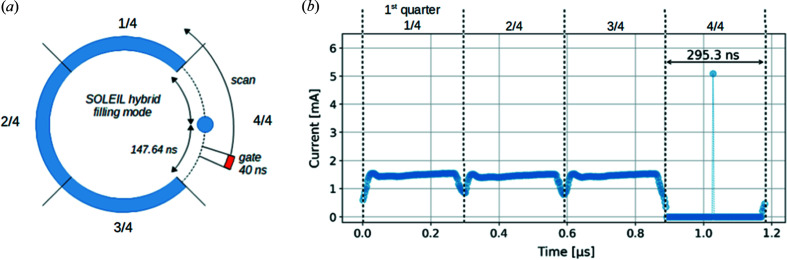
(*a*) Simplified diagram of the hybrid filling mode at Synchrotron SOLEIL and (*b*) the time distribution of current per electrons bunch.

To assess the advantages of this detector for time-resolved experiments, that is, experiments that require a short gate time, a series of tests aimed at isolation of the photons from a single-electron bunch were performed. During measurements the synchrotron was operated with hybrid filling mode and an isolated electron bunch occurred in the middle of the empty one-quarter of the storage ring, as illustrated in Fig. 11 ▸(*a*). The remaining three-quarters were filled with electron multi-bunches. The current distribution of electrons in this hybrid filling mode, measured with an electron beam monitor, is shown in Fig. 11 ▸(*b*). The time gaps between the isolated and multi-bunch sections are 147.64 ns at Synchrotron SOLEIL. The measurements consisted of mapping the storage ring filling mode by scanning the delay Δ*t* between the reference signal from the synchrotron and the detector gate. For every Δ*t* value, pixel counters were activated to count photons during a 40 ns interval. The final image was composed by accumulating the requested number of gates in the readout electronics. The filling-mode scan was carried out in two configurations, using the fluorescence and direct-beam setups described in Section 3.1.

**Figure 12:**
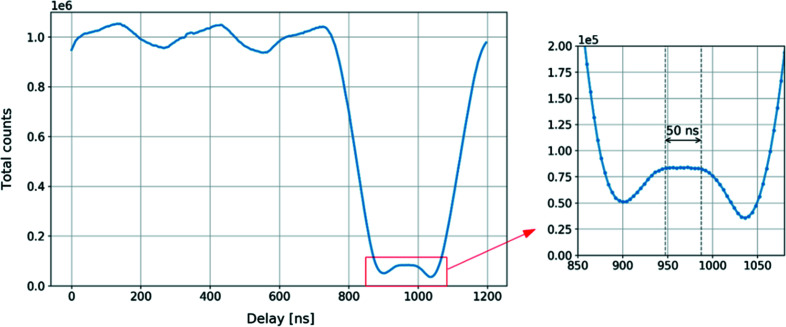
Hybrid filling mode measured with the XSPA-500k detector. The measurement was performed by illuminating the whole detector surface with Ge fluorescence radiation. For every delay value, 8000 frames of 40 ns were accumulated into a single image with all pixels summed. The estimated time gap during which the detector counts photons only from the isolated bunch is estimated to be 50 ns.

**Figure 13:**
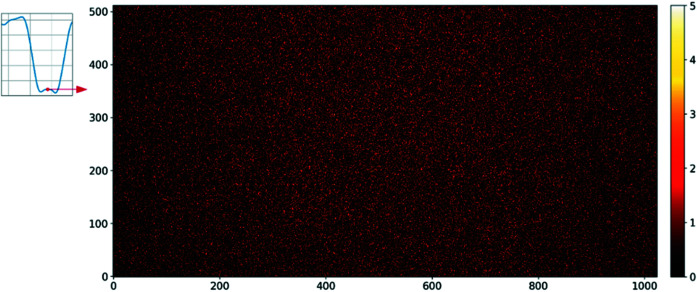
Flat-field image obtained from the isolated bunch photons at a delay of 980 ns. Although the image is highly granular due to the low statistics, the count distribution is uniform over the whole detector surface.

In the first case, the complete detector surface was illuminated with Ge fluorescence (9.9 keV). A complete scan was performed in the range from 0 ns to 1200 ns with a step of 4 ns. For every delay, 8000 gates were accumulated into a single image. The resultant filling mode profile is shown in Fig. 12 ▸. Due to the low photon count rate in the fluorescence configuration, every point on the plot represents the total signal (sum of all pixels) measured by the detector. The time window during which the detector counts photons only from the isolated electron bunch is estimated to be about 50 ns. Nevertheless, the curve cannot reach completely 0 counts between isolated and multi-bunch regions, due to the time resolution of the detector over the whole pixel matrix, which is affected by several factors, mainly gate signal propagation across the pixel matrix, but also the pulse width in a readout pixel (Kmon *et al.*, 2016 ▸; Koziol *et al.*, 2018 ▸). The image with counts from the single-bunch acquisition at a delay of 980 ns is shown in Fig. 13 ▸. The overall statistics on the image were low, producing a highly granulated pattern. However, it has been determined that, within a 50 ns time window, counts of successive images are comparable and uniformly distributed about the detector surface.

**Figure 14:**
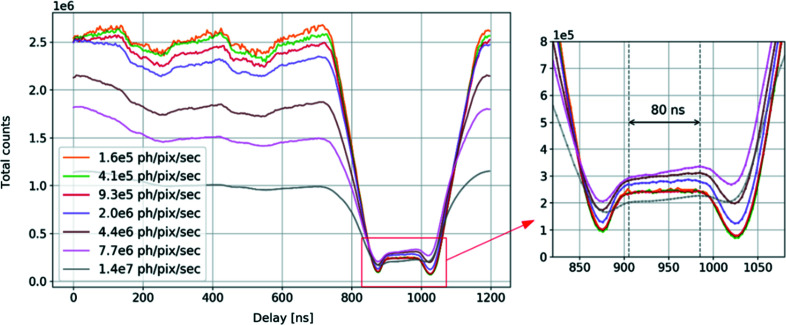
Hybrid filling mode measured with the XSPA-500k detector with a direct 9.9 keV beam with varied photon flux intensities. Every delay value represents the sum of all pixels accumulated in 1000 gates and corrected with a corresponding attenuation factor.

The same measurement was repeated using the direct-beam setup to verify the performance of the detector in single-bunch isolation mode while exposed to a much higher count rate. The same configuration as for the high-count-rate measurement (see Section 3.2.4) was used. The beam intensity was changed with a series of aluminium foils placed in the beam path. For every set of foils, the beam attenuation factor was measured with a calibrated photodiode. The filling mode scans were measured in the delay range from 0 ns to 1200 ns by accumulating 1000 gates of 40 ns, with a step of 4 ns. The resulting profiles, which represent the sum of all pixels after applying correction with the corresponding attenuation factor, are shown in Fig. 14 ▸. In the single-bunch region, the profiles obtained overlap for count rates up to 10^6^ photons s^−1^, which confirms the capability of the detector to isolate the bunch up to a relatively high photon flux and is in good agreement with counting rate linearity measurements. Above that beam flux, the pile-up effect starts to dominate in the multi-bunch section causing a decrease of the signal. At the same time, an increase of the signal is observed in the single-bunch region due to the fact that the detector is no longer able to isolate single-bunch photons and counts from the single and multi-bunch region start to overlap. The time window when the detector isolates the bunch accurately is about 80 ns, twice as long as that of the fluorescence measurement. This is due to the fact that in the case of the fluorescence measurement the whole detector is used, whereas in the direct-beam measurements only a small ROI of 35 × 36 pixels was considered, resulting in an improvement of the time resolution of the detector. Results obtained with a short gate time (see Fig. 14 ▸) demonstrate the excellent performance of the detector over the entire linear range, which is currently state-of-the-art performance for single-photon-counting detectors.

## 4. Conclusions   

A very fast and uniform HPC detector has been developed by Rigaku and AGH-UST. The detector was fully evaluated and characterized on the Metrology beamline at Synchrotron SOLEIL together by the SOLEIL Detector Group, AGH-UST and Rigaku. The results of the tests show MTF, NPS and DQE are near theoretical limits, an input count rate of about 10^7^ counts s^−1^ per pixel and a gate time on the order of 50 ns. These properties, along with the continuous readout up to 56 kframes s^−1^ and burst mode up to 970 kframes s^−1^ at 2-bits pixel^−1^, will push forward the lower limits of XPCS and time-resolved scattering experiments to nearly 1 µm per burst.

The hardware development of a single-module XSPA detector has been completed, and multi-module detectors such as XSPA-1M (two modules) and XSPA-4M (eight modules) are in development. The server software included with the detector is compatible with *EPICS* and will be adapted for use with *TANGO* in the near future.

These results show that the XSPA detector system will be a very powerful tool to explore new frontiers in X-ray science.
